# The effect of transcranial focused ultrasound target location on the acoustic feedback control performance during blood-brain barrier opening with nanobubbles

**DOI:** 10.1038/s41598-019-55629-2

**Published:** 2019-12-27

**Authors:** Bingbing Cheng, Chenchen Bing, Rajiv Chopra

**Affiliations:** 1 Department of Radiology, UT Southwestern Medical Center, Dallas, TX USA; 2 Advanced Imaging Research Center, UT Southwestern Medical Center, Dallas, TX USA; 3 Present Address: Department of Radiology, University of Calgary, Calgary, AB Canada

**Keywords:** Blood-brain barrier, Blood-brain barrier, Biomedical engineering, Biomedical engineering

## Abstract

Real-time acoustic feedback control based on harmonic emissions of stimulated microbubbles may be important for facilitating the clinical adoption of focused ultrasound (FUS)-induced blood-brain barrier (BBB) opening, both to ensure safe acoustic exposures, and to achieve repeatable and consistent opening. Previously our group demonstrated that successful BBB opening was achievable with both commercially available microbubbles and custom-made nanobubbles under acoustic feedback control. In a recent study, we demonstrated the acoustic control performance was not sensitive to the nanobubble concentration within 10^9^–10^11^ bubbles/ml. The goal of this study was to examine the effect of the ultrasound target location in the rat brain on the acoustic control quality during BBB opening with nanobubbles. Temporal analysis of the received acoustic signals during each ultrasound pulse indicated that stable nanobubble oscillation was present throughout the entire 10 ms ultrasound exposure. The acoustic feedback control signals were very sensitive to the brain spatial location in rats. There appears to be a shared pattern of acoustic control stability in the brain across different animals, suggesting anatomical features are an underlying cause. The findings emphasize the importance of tuning acoustic feedback control algorithms for specific rodent brain regions of interest to ensure optimal performance.

## Introduction

The use of focused ultrasound (FUS) to open the blood-brain barrier (BBB) has been investigated for many decades, but achieving consistency has always been challenging, and high intensities were required^1,2^. When combined with microbubbles (gas filled bubbles smaller than one hundredth of a mm in diameter, often used in life science, medical imaging, and industry), Hynynen *et al*. for the first time demonstrated reproducible, consistent, and local blood-brain barrier opening without obvious permanent tissue damage by using low-intensity focused ultrasound (16–690 W/cm^2^)^3^. Microbubbles dramatically reduced the ultrasound power required for BBB opening, and also concentrated the energy at the microvasculature improving the selectivity and success rate of BBB opening^3^. Since then, there has been a growth in the research activity investigating BBB opening using focused ultrasound with circulating microbubbles or sub-micron bubbles for efficient drug delivery into the brain for the treatment of brain disorders^4–19^. Recently, the safety of this technique has been evaluated in patients with various brain diseases in several clinical trials, e.g, tumor-bearing patients^17^, and patients with Alzheimer’s disease^7^. A good feasibility and safety profile have been demonstrated.

Despite this success, there are still challenges that need to be addressed to further facilitate the clinical adoption of this technique. One major requirement is the development of methods for real-time treatment monitoring and control of BBB opening. Due to the nature of anatomical differences in the brain from patient to patient (e.g. skull thickness variations or vascular pattern differences), it is not possible to transmit a fixed pressure into the brain to achieve the same therapeutic effect (e.g., delivering the same amount of drugs) across patients. Even within a single patient, the ultrasound pressure required to open the BBB in different brain locations will vary due to variations in skull anatomy and brain vasculature. To address this challenge, several groups including ours are investigating the utility of non-linear acoustic emissions from stimulated microbubbles as a feedback indicator to achieve safe and consistent BBB opening.

When applying focused ultrasound exposures, bubbles within the focal volume will oscillate at the driving frequency and harmonics. The strength of the harmonic oscillations is related to the pressure in the focal volume. As pressure increases, the bubbles oscillate in an increasingly non-linear manner. This non-linear oscillatory motion generates acoustic emissions at even, odd, and sub-harmonics of the fundamental driving frequency, which can be detected using a remote acoustic detector (cavitation detection). For an ultrasound transducer with a central frequency of f_0_, cavitation detection is usually performed on its harmonics (n × f_0_, n = 1, 2, 3…) or sub-/ultra-harmonics (n × f_0_, n = 0.5, 1.5, 2.5…). Several control algorithms based on these non-linear acoustic emissions have been developed^20–30^. Previous work by O’Reilly *et al*. minimized the adverse effects by identifying the pressure when ultraharmonic emissions were detected and then conducting all following sonications at a fixed percentage of this pressure^26^. Tsai *et al*. utilized subharmonic emissions as a control signal to predict BBB-opening occurrence^30^. By using a threshold subharmonic energy spectrum density change of 5.5 dB, they demonstrated improved sensitivity and specificity in discriminating successful BBB opening^30^. In a recent work by Sun *et al*., a closed-loop control algorithm based on harmonic emissions was developed and the ability to modulate the drug delivery dosage within a therapeutically effective range while improving safety control was demonstrated^21^. There are some shared features across these algorithms: (1) they are all based on monitoring 1 or more harmonic bands to adjust the ultrasound exposure level compared to a pre-defined threshold; (2) acoustic emissions outside the expected harmonic frequency bands are used as indicators of inertial cavitation which is believed to be associated with tissue damage; and (3) a bolus of microbubbles are administered into the bloodstream intravenously, which results in a time varying concentration during application of the control signal. This last characteristic creates a complicated time-varying control problem.

We recently developed a feedback control algorithm based on the first ultra-harmonic emissions of stimulated microbubbles by maintaining the ultra-harmonic emission at a threshold level for a fixed duration during an infusion of microbubbles. Successful BBB opening was demonstrated using this real-time controller in rodent models with both commercially available microbubbles (Optison: perflutren protein-type A microspheres injectable suspension, GE Healthcare, Waukesha, WI, USA; and Definity: perflutren lipid microsphere injectable suspension, Lantheus Medical Imaging, Inc., North Billerica, MA, USA) and a lab-made nanobubble^20,22^. Improved acoustic control performance was observed with nanobubbles (lipid bubbles with sizes range from 200–400 nm) in terms of the overall acoustic emission stability^20^. In a prior study, we have investigated the influence of nanobubble concentration on the acoustic control performance during BBB opening and demonstrated that the acoustic control performance was not sensitive to the nanobubble concentration within 10^9^–10^11^ bubbles/ml^22^. This current study aims to evaluate the sensitivity of acoustic feedback control performance to the spatial location of the acoustic focus within the rat brain during BBB opening with focused ultrasound and nanobubbles. The overall goal of these investigations is to develop a robust strategy for BBB opening in rodents to facilitate neuroscience research into the applications of this novel bioeffect.

## Results

### *In vivo* acoustic characterization of ultraharmonic emissions

**Figure 1:**
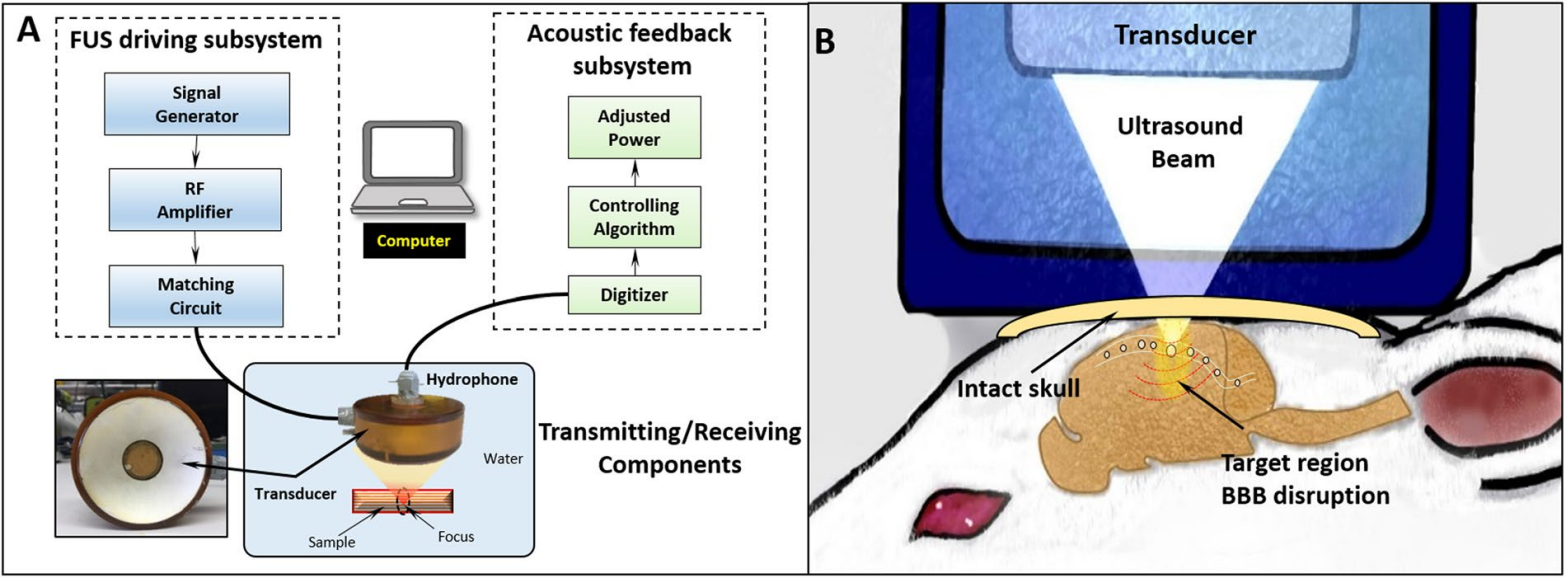
Acoustic feedback control ultrasound system. (**A**) The system components include an ultrasound transmitting subsystem and an acoustic feedback control subsystem. The ultrasound transmitting system is comprised of a signal generator, RF amplifier, impedance matching circuit, and a single element transducer; The feedback control system includes a hydrophone with a central frequency of 0.75 MHz, digitizer, and an algorithm which can adjust the ultrasound pressure based on the detected acoustic emissions from the stimulated bubbles. (**B**) Transcranial *in vivo* setup. Details of the system could be found in our previous publication^22^.

**Figure 2:**
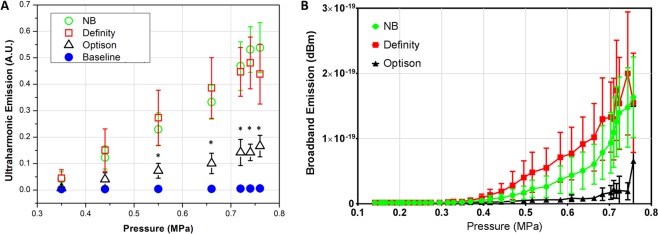
*In vivo* acoustic emissions. (**A**) *In vivo* AUC as a function of ultrasound pressure for nanobubble (n = 8), Definity (n = 6), Optison (n = 4), and baseline (n = 3). The linear relationship indicated it’s feasible to use ultraharmonic emissions as the control signal. (**B**) Broadband emission as a function of ultrasound focal pressure. Error bar: standard deviation (n = 8 for NB, n = 6 for Definity, n = 4 for Optison). *p < 0.05.

The system setup for this study is shown in Fig. 1 (see details in Materials and Methods). To evaluate the ultraharmonic emissions and broadband emissions of nanobubbles as a function of ultrasound pressure, *in vivo* characterizations were performed using three different agents (Fig. 2). Nanobubbles have a similar ultraharmonic response to that of Definity (p > 0.05 at all pressures) with the dilutions described in Materials and Methods, and both nanobubbles and Definity have significantly stronger ultraharmonic emissions than Optison under pressures above 0.44 MPa (p < 0.05), as shown in Fig. 2A. The linear regression coefficients (R^2^) of data for nanobubble, Definity, and Optison are 0.97, 0.99, and 0.99, respectively. The broadband emission of three bubbles due to inertial cavitation was also analyzed and the results are shown in Fig. 2B. In particular, little inertial cavitation was observed when the ultrasound pressure is below 0.5 MPa for nanobubbles.

**Figure 3:**
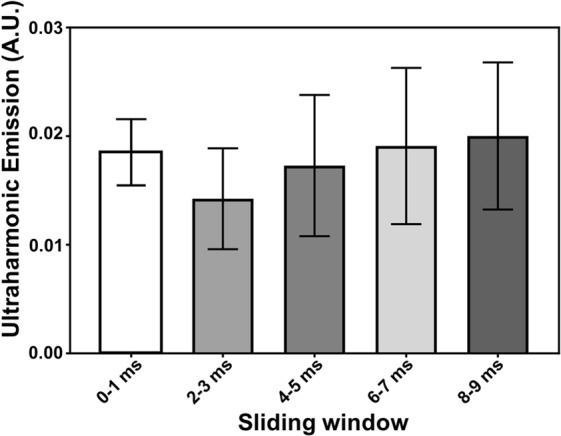
Ultraharmonic emission (AUCs) over a sliding window (bandwidth: 1 ms) on the recorded acoustic signals during the treatment. FFT was performed in the sliding window and the AUC was calculated in the frequency bandwidth 0.75 ± 0.05 MHz. Bars represent mean ± STD (n = 30). Stable cavitation was present throughout the ultrasound pulse.

The temporal dynamics of the received acoustic signal (9 ms) during each ultrasound pulse were analyzed by applying a sliding window (1 ms) and calculating the AUC (defined as the area under the curve of the frequency spectrum at 0.75 ± 0.05 MHz) within the window. Little to no signals in the broadband frequency (1.15 ± 0.0125 MHz) were observed in any of the selected windows. The results indicate a stable cavitation was present throughout a single ultrasound pulse and the whole treatment. Similar results were observed for all locations exposed in the brain. Figure 3 shows the results from a representative treatment at one brain location (30 pulses in the control phase). Each column represents the mean and standard deviation (std) of the AUC values (n = 30) within the selected time window.

### Sensitivity analysis of feedback control performance to brain location

**Figure 4:**
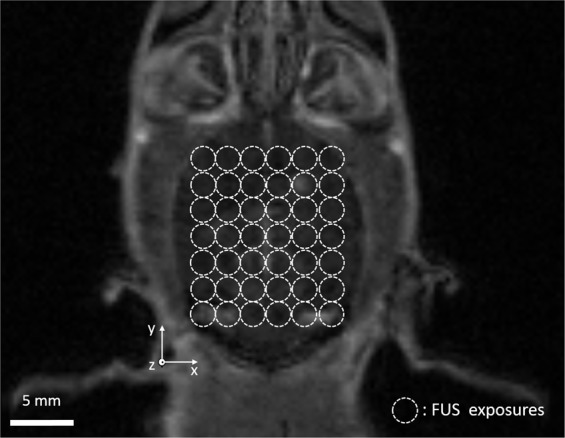
Treatment planning on a T1w MR image of a rat brain (transverse). The ultrasound exposures were applied in a 7 × 6 grid with 2 mm spacing to cover the majority of the brain. Conditions for each target: pulse length: 10 ms, pulse repetition frequency: 1 Hz, exposure duration: 30 s under real-time feedback control, and AUC control threshold: 0.05.

**Figure 5:**
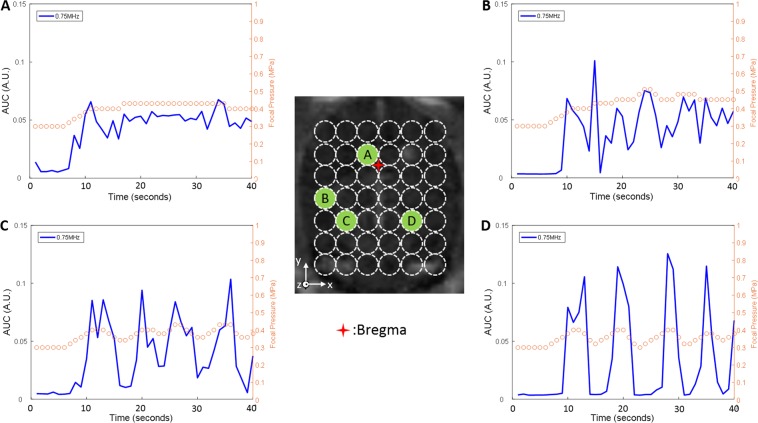
Typical AUC and pressure as a function of time in feedback control. (**A**–**D**) are from different brain locations indicating varied control performance. AUC control threshold was set to 0.05. The blue curves represent the acoustic emissions (AUC) as a function of time and the orange circles represent the ultrasound focal pressure adjusted in real-time to maintain the AUC at a desired level.

Treatment planning was performed based on T1-weighted MR images acquired beforehand. To cover most part of the brain, 42 (7 × 6) locations were assigned at one x-y plane of the rat brain. The locations were circled out in Fig. 4. At each location, ultrasound exposures were applied under real-time feedback control. The acoustic emissions from stimulated nanobubbles were recorded and analyzed and the results are shown in Fig. 5. The control performance varied among different spatial locations of the brain. The control at location A (Fig. 5A) was stable and the AUC was well maintained at the target AUC level (0.051 ± 0.008). The ultrasound pressure was almost constant except the initial ramp-up phase (0.41 ± 0.01 MPa). However, at location D (Fig. 5D), the control signal was much noisier (0.041 ± 0.044).

**Figure 6:**
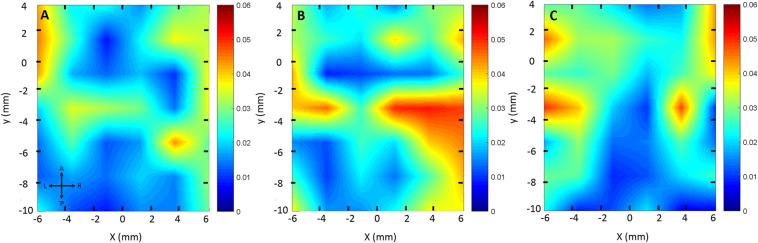
Control stability map as a function of spatial location in the x-y plane. (**A**) The first animal; (**B**) the second animal; (**C**) the third animal. The color map represents the standard deviation (std) during feedback control at each location. Smaller std (blue) represents a more stable control performance, while larger std (red) shows relatively a less stable control performance. The map was interpolated using a linear method. x = 0, y = 0 indicates Bregma.

**Figure 7:**
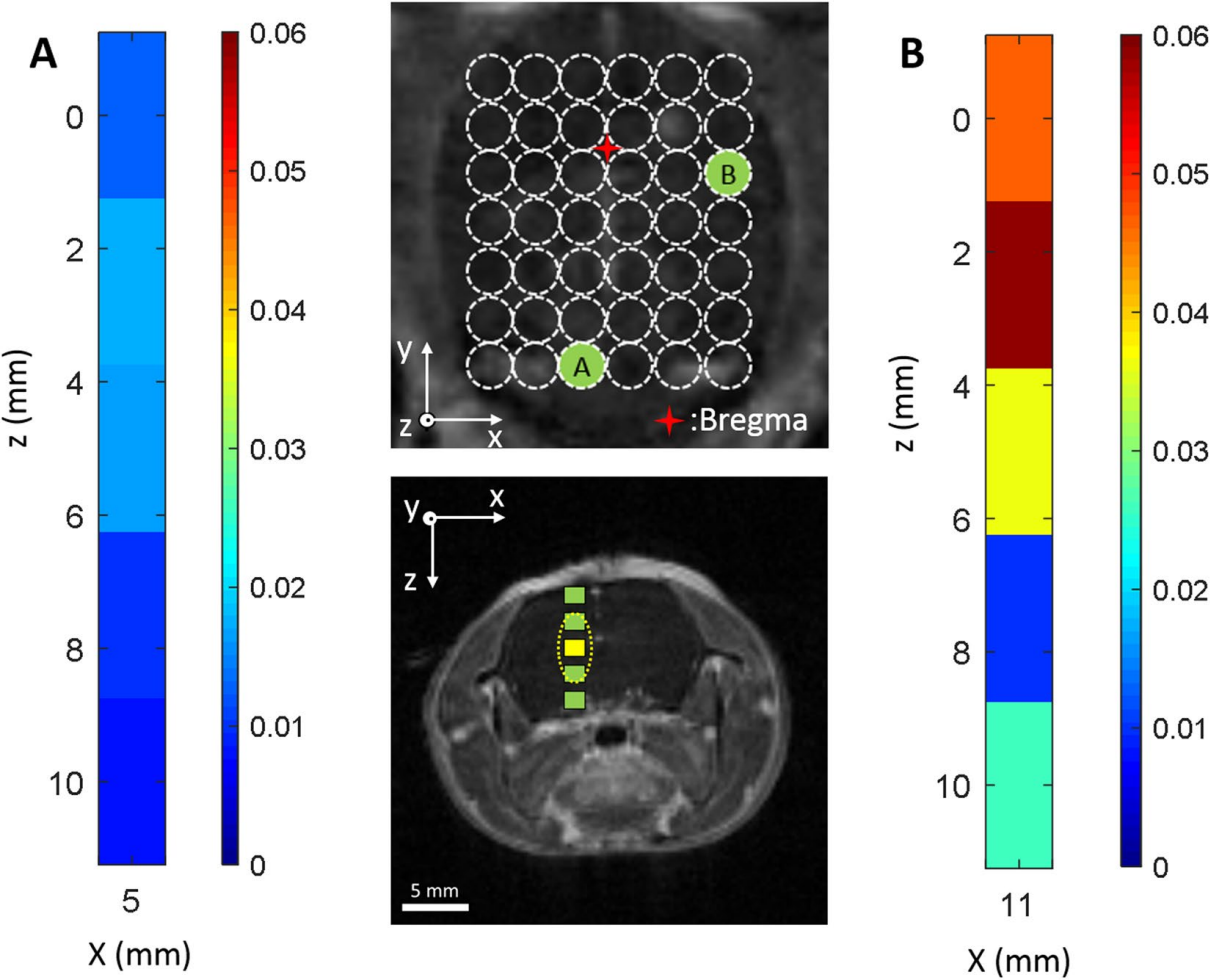
Control stability map as a function of spatial location in the x-z plane. (**A**,**B**) Control stability in vertical direction at different locations in x-y plane. The top middle panel shows the corresponding locations in x-y plane and the panel below that indicates the five scanning points. Two locations (**A**,**B**) in the x-y plane were selected. One was from posterior brain region and the other one was on the top right edge. The center of ultrasound focus was indicated by the green squares and the ultrasound focal beam was indicated by the dashed elliptical circle as an example. Ultrasound beam at different z locations have some overlap due to the nature of elongated axial ultrasound focus.

By calculating the standard deviation of the acoustic signals acquired during feedback control, we were able to generate a color map in which the relationship between control stability/sensitivity and brain locations is shown. Figure 6 shows three control sensitivity maps with regards to brain spatial locations in the horizontal plane from three animals. The posterior brain (y < −6mm) tends to have a stable control performance (std < 0.02, z = 4 mm) while the left and right edges along the y direction tend to have a less stable control (std > 0.03, z = 4 mm). The effect of spatial location in vertical (z) direction was also evaluated and shown in Fig. 7. As Fig. 7 shown, the control performance was not sensitive to the z location in regions where good control was identified in the x-y plane (Fig. 7A). On the contrary, in regions (x-y plane) where unstable control occurred, the acoustic feedback signal was sensitive to the z location (Fig. 7B).

**Figure 8:**
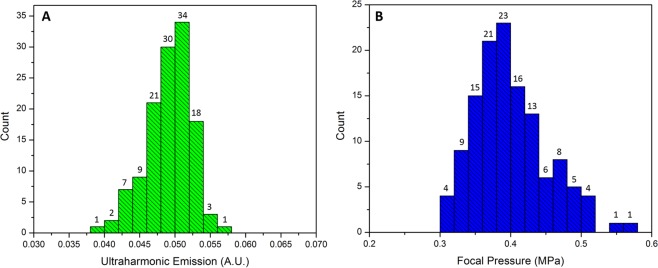
Histogram of mean ultraharmonic emissions (**A**) and mean ultrasound focal pressures (**B**) during feedback control. The total number of exposures are 126, across 3 animals.

Figure 8A,B show the histograms of mean values of the ultraharmonic emissions and ultrasound pressures during feedback control in each sonication across 126 targets. Ninety-one percent (115/126) of mean AUCs with a control threshold of 0.05 fell within 0.05 ± 0.005 (Fig. 8A). More than half of the mean AUCs (56%) fell in the left side of 0.05 while eighty-three percent were less than 0.0525, indicating this controller has a good ability to maintain the nanobubble activity in a less aggressive/safer manner. Ninety-five percent (120/126) of mean ultrasound pressures were less than 0.5 MPa during feedback controlled BBB opening treatment (Fig. 8B).

## Discussion

Acoustic cavitation was found to be attributed to FUS-induced BBB opening through stable and inertial cavitation^31^. It is assumed that successful BBB opening without damage is induced by stable cavitation, and inertial cavitation can be used as an indicator of potential tissue damage^31,32^. For an ultrasound transducer with a central frequency of f_0_, cavitation detection is usually performed on its harmonics (n × f_0_, n = 1, 2, 3…) or sub-/ultra-harmonics (n × f_0_, n = 0.5, 1.5, 2.5…). The former represents the acoustic response from both tissue and bubbles while the latter is the unique acoustic response from stimulated bubbles through nonlinear oscillation^33^. Therefore, our controller was mainly focused on the ultra-harmonic signals. The highly linear relationship between the ultra-harmonic emissions and the acoustic pressure within a certain range across three evaluated bubbles (Fig. 2A) supported that it is feasible to use ultra-harmonics for feedback control^21^. Persistent stable cavitation of nanobubbles was observed throughout the entire ultrasound exposure (10 ms) when using our real-time controller. This was supported by the results of the dynamic analysis of the received acoustic signals during feedback control (Fig. 3). In addition, no significant decrease was observed when comparing the results from the first pulse to the last pulse in the 30 s treatment at any selected window as the error bar shows. This was believed to be attributed to the bubble infusion instead of a bolus injection (more consistent bubble concentration in the bloodstream).

Real-time acoustic feedback control may be important for facilitating the clinical adoption of FUS-induced BBB opening, both to ensure safe acoustic exposures, and also to achieve repeatable and consistent opening across different brain regions. Our results indicate the acoustic feedback control signal is sensitive to the target brain location in the rats, especially in the transverse plane (x-y plane). The backscattered acoustic signals from stimulated nanobubbles showed significant variations across different brain locations (Blue lines in Fig. 5). Although the controller was able to maintain the mean AUC at the target level (Fig. 8A), the stability (e.g., AUC standard deviation) varied significantly from location to location (Figs. 6–7). There appears to be a shared pattern among the control stability maps across three animal brains (Fig. 6). Note that there might be some offset (~mm) of the sonicated brain locations across different animals due to the manual lambda and bregma registration process. In general, the middle brain in the anterior region and the majority of the posterior region have a more stable control (AUC std ≤0.02 while targeting at 0.05). The variability of the received acoustic signals in left and right edges tends to be higher, which requires extra attention when performing feedback controlled FUS-BBB opening in those regions. As shown in Fig. 5D, the signal in this location was very sensitive to small pressure variations, making stable control difficult. In the regions (x-y plane) where good control performance was observed, the acoustic signal was not sensitive to the z location. However, in the less stable control regions (e.g., location B on the right edge in Fig. 7), the control signal was sensitive to the z location even though the ultrasound beams were partially overlapped with each other. The result in Fig. 7B also suggests that even in a bad control region (x-y plane), one can still bring the control stability in the range of the good location by adjusting the depth.

A limitation of this study is that the underlying mechanism for these variations in acoustic emissions was not addressed. However we can identify several possibilities for further study. First, the vascular pattern of large vessels in the brain may contribute to the variation in acoustic emissions. The pattern in control stability maps shown in Fig. 6 appears to be similar to the large vein distribution in the rat brain^34^. Second, the skull geometry (e.g., curvature and/or thickness) may also play a role in the variation of acoustic emissions during feedback control. The different skull thickness at different locations would have led to variations in the mean ultrasound pressure required, but not necessarily in the control stability. However, when combined with the skull curvature, they might contribute to the large acoustic emission variability at the left and right edges of the brain, assuming a bigger curvature was present there. Lastly, it’s possible that there were different levels of standing waves (a wave which oscillates in time, but the peak amplitude profile does not move in space) at different brain locations, which caused variability. This may be partially supported by the result in Fig. 7B. By moving the ultrasound focus up and down, one may be able to avoid the standing waves and achieve a good control. Previously, O’Reilly *et al*. reported that at a similar ultrasound frequency (~0.5 MHz), the location of the occurrence of standing waves as well as the amplitude of the wave was impacted by the sonication location in the skull^35^. In that study, although they didn’t investigate the full spectrum of brain locations, they observed two different ultrasound focal pressure profiles at two brain locations. They also determined that the hydrophone measurements were strongly sensitive to the geometry of the skull.

Another limitation of this study is the BBB opening extent was not comprehensively evaluated at different brain locations. In a preliminary study, we demonstrated the successful BBB opening was achievable using the parameters in this work (10 ms pulse length, 1 Hz PRF, and 30 s duration under feedback control at AUC = 0.05, see Fig. S1). In this study, we identified that the control performance is sensitive to the brain location, however, its influence on the BBB opening such as BBB opening time, drug delivery efficiency was not fully evaluated. Further studies are warranted to establish a link between acoustic control quality and BBB opening.

## Conclusion

In this study, we demonstrated that it is feasible to use ultraharmonic emissions as the feedback control signal for commercially available microbubbles and customized nanobubbles. With nanobubbles, stable cavitation was present throughout the entire 10 ms ultrasound burst. We also demonstrated that acoustic feedback control stability was dependent on the spatial brain location being targeted, with similar regions of good and poor performance across multiple animals. These findings emphasize the importance of tuning acoustic feedback control algorithms for specific rodent brain regions of interest to ensure optimal performance.

## Materials and Methods

### Materials

Materials for nanobubble synthesis are same as described in a previous study^22^. Lipids 1,2-dibehenoyl-sn-glycero-3-phosphocholine (DBPC), 1,2 Dipalmitoyl-sn-Glycero-3-Phosphate (DPPA), and 1,2-dipalmitoyl-sn-glycero-3-phosphoethanolamine (DPPE) were purchased from Avanti Polar Lipids (Alabaster, AL, USA). 1,2-distearoyl-snglycero-3-phosphoethanolamine-N-[methoxy(polyethyleneglycol)-2000] (ammonium salt) (mPEG-DSPE) was obtained from Laysan Lipids (Arab, AL, USA). Pluronic L10 was obtained from BASF Corporation (Florham Park, NJ, USA) and glycerol was purchased from Thermo Fisher Scientific (Waltham, MA, USA). All chemicals were used directly without further purification. Octafluoropropane (C_3_F_8_) gas was obtained from FluoroMed, L.P. (Round Rock, TX, USA).

### Nanobubble synthesis

The nanobubble synthesis protocol was adopted from our previous reports^20,22^, and is based on a formulation described originally by Krupka *et al*.^36^. Briefly, the nanobubbles were prepared by mixing a variety of lipids: DBPC, DPPA, DPPE and mPEG-DSPE with a mass ratio of 6.15:1:2:1 in chloroform in a 1 ml vial^37^. Following solvent evaporation, hydration and gas exchange (to octafluoropropane), the bubbles were activated via mechanical agitation and centrifugation was performed to eliminate larger bubbles over 1 micron in size.

### Ultrasound system and feedback control algorithm

The stereotactic focused ultrasound system used in this study was the same as described in our previous reports as shown in Fig. 1A^20,22^. Briefly, a custom-made single-element focused PZT transducer with a 75-mm diameter and a 60-mm radius of curvature (DL-54, DeL Piezo Specialties, LLC, West Palm Beach, FL, USA) was used to transmit ultrasound energy into the brain. The fundamental anti-resonant frequency of the transducer was 0.5 MHz as measured with an impedance analyzer (C-60, Cypher Instruments, London, UK). The spatial pressure distribution at the transducer focus and the pressure-voltage relationship were characterized in a hydrophone tank with a needle hydrophone (SN2344, Precision Acoustic, Dorchester, Dorset, UK). A 3D-printed cone filled with degassed water was attached to the transducer and enabled coupling of the ultrasound beam to the rat brain. The transducer was connected to a custom-built driving system and stereotaxic apparatus (51730 M, Stoelting Co., Wood Dale, IL, USA). A hydrophone which was constructed from a flat 20-mm diameter PZT composite material (DL-53 1-3 composite, center frequency 0.75 MHz, DeL Piezo Specialties, LLC, West Palm Beach, FL, USA) was inserted into a 26-mm diameter aperture in the center of the transducer to detect the stimulated acoustic emissions of micro- or nano- bubbles. The signal acquired by the hydrophone was sampled at 20 MHz using a 14 bit PCI digitizer (ATS460, Alazar Technologies Inc, Pointe-Claire, QC, Canada).

During each ultrasound exposure (10 ms burst, 1 Hz repetition frequency), acoustic emissions detected with the hydrophone were digitized and the frequency spectrum was calculated using the Fast-Fourier transform. The area under the curve (AUC) was calculated by summing up the amplitude at the ultra-harmonic frequency (0.75 MHz) within a 100 kHz bandwidth. During feedback control, the average AUC value from the last three acquisitions was compared with a desired AUC threshold and the pressure for the next ultrasound exposure was adjusted accordingly using a step size of 0.01–0.03 MPa.

### Animal preparation

Female rats (Sprague Dawley, 230–300 g, n = 14) were used in this study. All procedures were approved by the UT Southwestern Institutional Animal Care and Use Committee and followed guidelines set forth by the Guide for the Care and Use of Laboratory Animals. Ten animals were used in pressure sweep experiments (NB: n = 2, 4 targets/rat; Definity: n = 3, 2 targets/rat; Optison: n = 2, 2 targets/rat; Baseline: n = 3, 1 target/rat). Four animals were used in the spatial brain scan using ultrasound exposures suitable for BBB opening (for x-y plane: n = 3, 42 targets/rat; for x-z plane: n = 1; 10 targets/rat). Only nanobubbles were used when evaluating the stability of feedback control at different brain locations as it’s the primary goal of this study. Animals were anesthetized through inhalation using 2–3% isoflurane. A 24 G I.V. catheter was placed in the lateral tail vein for drug administration. A physiologic monitoring system (PhysioSuite, Kent Scientific Corp., Torrington, CT, USA) was used to monitor vital signs and maintain core body temperature throughout the experiment. Hair over the cranial surface of the skull was removed using an animal clipper and depilatory cream (VEET sensitive formula, Reckitt Benckiser, Parsippany, NJ, USA) to enable ultrasound propagation into the brain. After preparation, the animal was transferred to the stereotaxic apparatus and stabilized using ear bars and a bite bar. A custom-built nose cone was placed over the animal’s nose to deliver inhalant anesthetic during sonications.

A small skin incision was made over the skull to identify cranial landmarks for atlas registration. The registration procedure was performed using the method described previously^16^. The ultrasound setup for the animal study is shown in Fig. 1B. Ultrasound gel was applied on top of the skin incision to allow the penetration of ultrasound beam. A water tank filled with degassed water was adapted to the stereotaxic apparatus for coupling. After treatment, animals were sacrificed using transcardiac perfusion with saline and 10% buffered formalin approximately 5–10 minutes after sonication. The brain was harvested immediately and placed in formalin for final fixation.

### Ultrasound exposures

For pressure sweep sonications, nanobubbles (custom-made with 1:10 dilution, 10^10^ bubbles/ml)^22^, Definity (Lantheus Medical Imaging, Billerica, MA, USA, 6 µl/kg with 1:100 dilution), or Optison (GE Healthcare, Milwaukee, WI, USA, 30 µl/kg with 1:20 dilution) were administered through the tail vein catheter with an infusion injection (0.3 ml/min). The dilutions of Definity and Optison were chosen to achieve a similar total gas volume to the original nanobubbles. Three acquisitions were acquired at each pressure level starting from 0.35 to 0.76 MPa. The duration of each pressure sweep sonication was approximately 125 seconds. The pressure is estimated based on the skull insertion loss at the focal location^38^.

For feedback-controlled FUS sonication at different locations, nanobubbles (10^10^ bubbles/ml) were infused at a rate of 0.1 ml/min through the tail vein catheter to avoid exceeding the maximum injection volume limit^39^. The target location inside the brain was defined using three coordinates (x, y, z) along medial-lateral (ML), anterior-posterior (AP), and dorsal-ventral (DV) direction. The ultrasound beam was perpendicular to the transverse plane of the brain. First, an x-y plane across the ultrasound beam was selected at DV (z) = 4 mm to investigate the feedback control stability at targets with different ML (x) and AP (y) coordinates. A raster scan was performed to cover the majority of the brain (10 × 12 mm^2^) in a 7 × 6 grid manner with a spacing of 2 mm. In total, 42 targets were treated. At each target, a 30 sec ultrasound exposure (10 ms burst, 1 Hz repetition frequency) was performed by controlling the ultra-harmonic emissions of nanobubbles at a desired threshold (AUC = 0.05).

A secondary scan was performed along the DV direction (z) to evaluate the feedback control quality at various depth with given ML and AP coordinates. Based on the initial ultrasound exposures in the x-y plane, two locations with good and less stable control performance were selected (AP = −7, ML = −1; AP = −1, ML = 5). For each location, the scan was performed at 5 different DV coordinates with a 2 mm spacing, respectively.

### Data analysis of received acoustic signals

During ultrasound exposures, the acoustic signals received via the hydrophone were converted to frequency spectrum and the AUC for the ultra-harmonic of 0.75 ± 0.05 MHz was calculated. The total power of the emission signal centered at 1.15 MHz with a bandwidth of 25 kHz was calculated as an indicator of broadband emission. For the brain scanning experiment, at each target, the mean and standard deviation of AUCs were calculated. Then a heatmap of AUC standard deviations at different spatial locations were generated in Matlab (R2016a, Mathworks, Natick, MA, USA). For the map in the x-y plane, a linear interpolation was performed to the heatmap by repeatedly dividing the intervals between points of the refined grid five times in each dimension. To further evaluate the dynamics of acoustic emissions at the ultra-harmonic frequency (0.75 ± 0.05 MHz) during the 10 ms ultrasound exposure, an analysis was conducted by moving a sliding window (1 ms width) on the 9 ms time domain signal and calculating the corresponding AUC values.

### Statistical analysis

Student t-test with Holm-sidak correction for multiple comparisons was performed in the *in vivo* acoustic characterization of ultraharmonic emissions using GraphPad (Version 3.1, San Diego, CA, USA). A p value less than 0.05 was considered significant.

## Supplementary information

Supplementary information
