# Supplementary material for: The effect of transcranial focused ultrasound target location on the acoustic feedback control performance during blood-brain barrier opening with nanobubbles
Source: Sci Rep. 2019 Dec 27;9:20020. doi: 10.1038/s41598-019-55629-2 (PMC6934715; doi:10.1038/s41598-019-55629-2)
Supplement: Supplementary file 1 — Supplementary information [file 41598_2019_55629_MOESM1_ESM.pdf]

The effect of transcranial focused ultrasound target location on the acoustic feedback control performance during blood-brain barrier opening with nanobubbles

Bingbing Cheng[1†], Chenchen Bing[1†], and Rajiv Chopra[1,2*]

[1] Department of Radiology, UT Southwestern Medical Center, Dallas, TX, USA; and [2] Advanced Imaging Research Center, UT Southwestern Medical Center, Dallas, TX, USA;

[†]: Current address: Department of Radiology, University of Calgary, Calgary, AB, Canada

[*]: Corresponding author: Rajiv Chopra, Email: rajiv.chopra@utsouthwestern.edu. Department of Radiology, University of Texas Southwestern Medical Center, 5323 Harry Hines Blvd, Dallas, TX, USA 75390

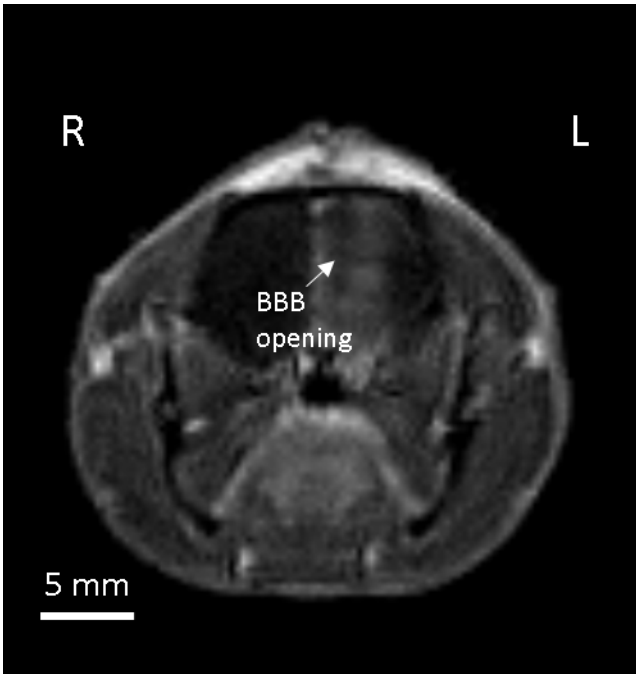

Figure S1. Contrast-enhanced T1w MR image shows the BBB opening in a rat brain with the following FUS parameters: 10 ms burst, 1 Hz repetition frequency, and 30s duration under feedback control at AUC = 0.05.
